# Drug-dependent functionalization of wild-type and mutant p53 in cisplatin-resistant human ovarian tum3or cells

**DOI:** 10.18632/oncotarget.14228

**Published:** 2016-12-26

**Authors:** Michelle Bhatt, Cristina Ivan, Xiaolei Xie, Zahid H. Siddik

**Affiliations:** ^1^ Department of Experimental Therapeutics, The University of Texas MD Anderson Cancer Center, Houston, Texas, USA; ^2^ Center for RNA Interference and Non-Coding RNAs, The University of Texas MD Anderson Cancer Center, Houston, Texas, USA

**Keywords:** cisplatin resistance, p53 tumor suppressor, missense mutation, Chk2, MAPK

## Abstract

Cisplatin (cis-Pt) resistance in tumor cells from p53 dysfunction is a significant clinical problem. Although mutation can inhibit p53 function, >60% of p53 mutants retain normal function according to literature reports. Therefore, we examined the status of p53 in cisplatin-resistant ovarian tumor models and its functional response to cis-Pt and the mechanistically-distinct non-cross-resistant oxaliplatin (oxali-Pt). Relative to sensitive A2780 cells harboring wild-type p53, the 2780CP/Cl-16, OVCAR-10, Hey and OVCA-433 cell lines were 10- to 30-fold resistant to cis-Pt, but was substantially circumvented by oxali-Pt. Mutant p53 in 2780CP/Cl-16 (p53^V172F^) and OVCAR-10 (p53^V172F^ and p53^G266R^) cells, predicted as non-functional in p53 database, displayed attenuated response to cis-Pt, as did the polymorphic p53^P72R^ (functionally equivalent to wild-type p53) in HEY and OVCA-433 cell lines. However, p53 was robustly activated by oxali-Pt in all cell lines, with resultant drug potency confirmed as p53-dependent by p53 knockout using CRISPR/Cas9 system. This p53 activation by oxali-Pt was associated with phosphorylation at Ser20 by MEK1/2 based on inhibitor and kinase studies. Cis-Pt, however, failed to phosphorylate Ser20 due to downregulated Chk2, and its clinical impact validated by reduced overall survival of ovarian cancer patients according to TCGA database. In conclusion, cis-Pt resistance occurs in both wild-type and mutant p53 ovarian cancer cells, but is associated with loss of Ser20 phosphorylation. However, these mutant p53, like polymorphic p53, are functional and activated by oxali-Pt-induced Ser20 phosphorylation. Thus, the potential exists for repurposing oxali-Pt or similar drugs against refractory cancers harboring wild-type or specific mutant p53.

## INTRODUCTION

Ovarian cancer is the leading cause of death from gynecological cancers among women. In 2015 over 21,000 women in the United States were diagnosed with ovarian cancer, of whom ~67% succumbed to their death [1]. The majority of ovarian cancer patients are diagnosed at an advanced stage, when the tumor has metastasized, resulting in a 5-year survival rate below 30% [2]. The current treatment for advanced ovarian carcinoma involves cytoreductive surgery in order to remove the bulk of the tumor, and since advanced ovarian cancer cannot be eliminated by surgery alone, patients also receive a combination of a platinum (Pt) drug, either cisplatin (cis-Pt) or carboplatin, with a taxane. Initially, patients exhibit a satisfactory response; however, about 80% of patients eventually develop resistance to therapeutic drugs resulting in the low survival rate [3, 4]. Therefore, in order to advance Pt-based therapy and enhance ovarian cancer responses in the clinic, studies that identify mechanisms of Pt resistance are pertinent [5].

The tumor suppressor p53 plays an important role in facilitating favorable antitumor drug response to Pt drugs. Under basal conditions, p53 is negatively regulated by binding to the inhibitors Mdm2 and Mdm4, which promote its proteosomal degradation. DNA damage by cis-Pt or carboplatin upregulates key cellular pathways to stabilize p53 by dissociating the Mdm2-Mdm4-p53 complex, thereby allowing p53 to translocate to the nucleus, where it binds to specific DNA sequences for transactivation of target genes, exemplified by p21, Mdm2 and Bax [6–8]. This transcriptional activity is essential for p53-dependent cellular effects, such as cell cycle arrest, senescence, and programmed cell-death [9–11]. Therefore, failure in p53 function has been identified as a significant mechanism contributing to Pt drug resistance. Factors reported to disrupt wild-type p53 function include i) overexpression of negative regulator Mdm2 or Mdm4, and ii) downregulation of kinases involved in post-translational regulation of p53 [12]. However, the frequency of Mdm2 or Mdm4 overexpression is low (2-4%) in ovarian tumor cells and cancer patients [13] or non-existent in cis-Pt-resistant ovarian tumor cell lines [14]. Therefore, it is possible that failure in post-translational phosphorylation of p53 may be the more important in Pt resistance, but very little has been reported. Normally, cis-Pt upregulates ATR, Chk1 and Chk2 kinases, which stabilize and activate p53 by phosphorylating Ser15 and Ser20, which are considered to be critical sites as they are located in the region of p53 that binds to Mdm2 [15–17]. Of these kinases, contribution of Chk2 to cis-Pt resistance is possible as defects in this kinase and p53 are reported to be mutually exclusive [18] and Chk2 dysfunction is known to exist in several cancers [7], including 23% of clinical ovarian cancer cases [19].

Another major cause of p53 dysfunction is mutation in the p53 gene [20], and several clinical studies have attempted to correlate p53 gene status with chemotherapy response [21–23]. However, the results have been conflicting, since wild-type or mutant p53 can be associated with both antitumor therapeutic response and resistance. Surprisingly, in high grade serous ovarian cancer (HGSOC), wild-type p53 is associated with a significantly inferior overall survival of patients [24]. These reports indicate that there is insufficient knowledge of how wild-type p53 is inactivated and whether all p53 mutations negatively impact p53 function and downstream cellular processes. However, one detailed study using the yeast functional assay (FASAY) for evaluating transcriptional activity of 2,314 p53 mutants revealed that only 9.6% exhibited no activity, 26.5% had partial activity and 63.9% expressed full activity relative to wild-type p53 [25]. These findings demonstrate that not all p53 mutants are inactive or dysfunctional and, therefore, classification of p53 function goes beyond the ‘wild-type *versus* mutant’ genotypic correlation. More importantly, we have previously demonstrated that in a cis-Pt-resistant ovarian tumor model harboring mutant p53, ionizing radiation, but not cis-Pt, induced and activated p53 [26]. In the present study, therefore, we have evaluated the response of wild-type or mutant p53 and its post-translational phosphorylation in well-studied models of cis-Pt resistance [27]. Since oxaliplatin (oxali-Pt) is known to circumvent cis-Pt resistance [28], this agent was also investigated to examine if its mechanism of action is linked to the mechanism of cis-Pt resistance. Our study indicates that Chk2 dysfunction is prevalent in cis-Pt-resistant cells, and the resultant loss in Ser20 phosphorylation is an important negative regulator of p53 function, both in wild-type and mutant p53 tumor cells. However, phosphorylation of this site is restored by oxali-Pt in a Chk2-independent manner to activate p53 and circumvent cis-Pt resistance in all tumor models, and this suggests loss of p53 phosphorylation, and not mutation, is the main driver of cis-Pt resistance in these models of ovarian cancer.

## RESULTS

### Response of cell lines and ovarian cancer patients based on p53 status

A number of cis-Pt-resistant ovarian tumor models have been reported, but based on our previous experience [26, 27], 2780CP/Cl-16, OVCAR-10, HEY and OVCA-433 were selected for the investigation. In addition, the A2780 cell line was included as a sensitive ovarian model, and to provide a matching pair to 2780CP/Cl-16 cells that were derived from A2780 cells [26]. Although the A2780 and 2780CP/Cl-16 cells are of ovarian origin, their histological sub-type is unknown, whereas OVCAR-10 is reported as being an adenocarcinoma and Hey and OVCA-433 as serous ovarian cancer [29]. Gene sequence analysis has confirmed that A2780 cells harbor wild-type p53, which is inducible and makes this cell line widely used as a cis-Pt-sensitive model of ovarian cancer [26]. In contrast, the resistant models demonstrated changes in amino acid sequence of p53 (Table 1). However, the P72R polymorphism in Hey and OVCA-433 models, as expected from the literature [20], does not inhibit transcriptional activation of target gene promoters in the yeast FASAY system (http://p53.fr). On the other hand, the V172F and G266R mutants appear to lack p53 function in this system.

**Table 1:** Analysis of p53 mutation and functional status in ovarian cancer cell lines

Cell Line	Histological Sub-Type^1^	Base Change	Zygosity Status	AA Change^2^	Gene Status	Function Status^3^
A2780	Unknown	None	None	None	Wild-type	+
2780CP/Cl-16	Unknown	139G>K	Heterozygous	V172F	Mutant	-
OVCAR-10	Adeno-carcinoma	139G>K, 14G>R	Heterozygous, Heterozygous	V172F, G266R	Mutant, Mutant	-
HEY	HGSOC	119C>G	Homozygous	P72R	Polymorphic	+
OVCA-433	HGSOC	119C>G	Homozygous	P72R	Polymorphic	+

^1^[29]

^2^AA, amino acid

^3^The p53 transcriptional activity (or p53 function) is predicted using a database of mutant p53 function (http://p53.fr/TP53Mutload/database_access/search.php); +, functional; -, inactive.

**Figure 1:**
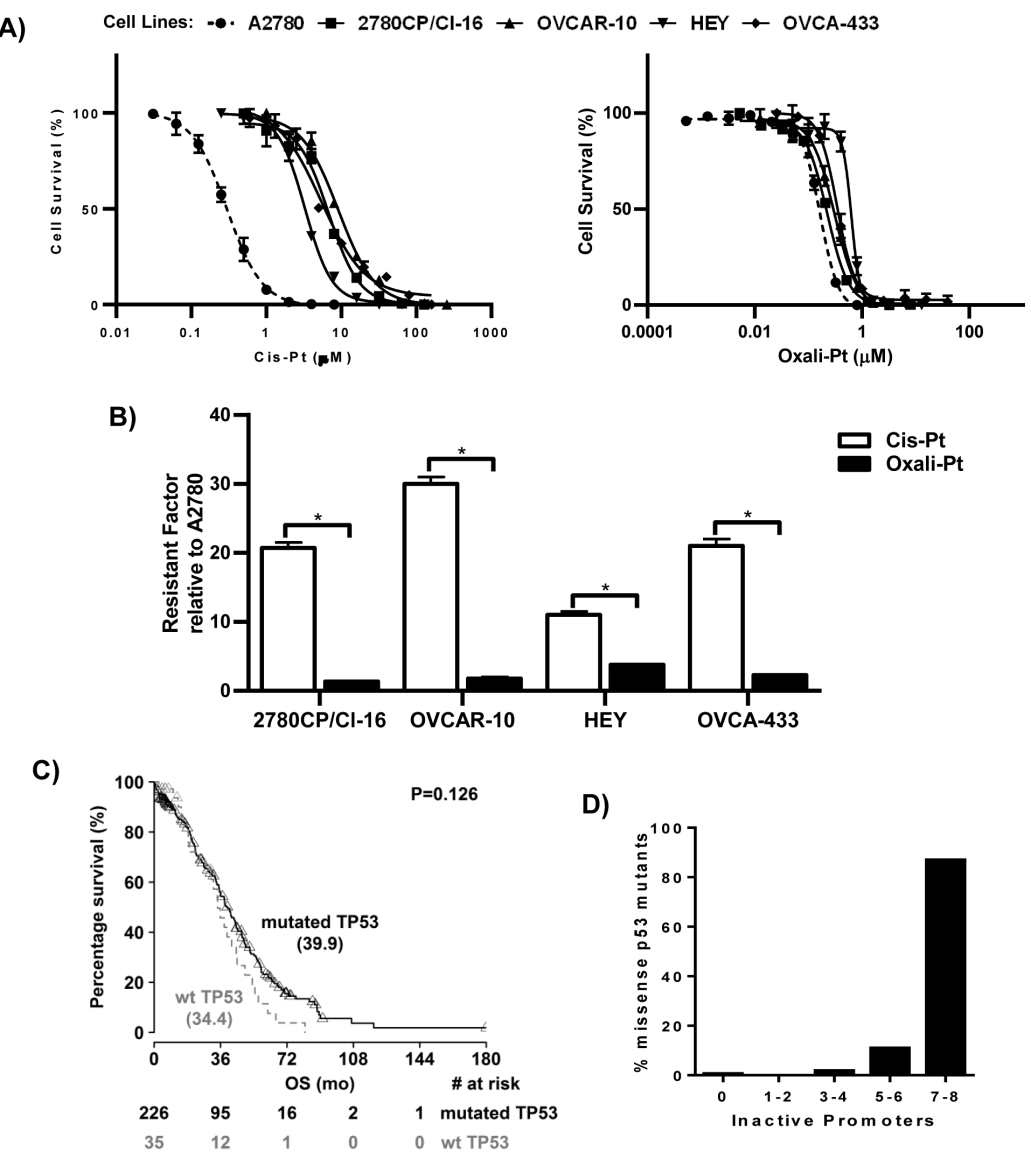
Cytotoxic drug response in cell lines and overall survival of patients based on p53 status **A**. Dose-response curves for cis-Pt and oxali-Pt in A2780, 2780CP/Cl-16, OVCAR-10, HEY and OVCA-433 cell lines using the 5-day MTT assay. **B**. Resistance factors of 2780CP/Cl-16, OVCAR-10, HEY and OVCA-433 cell lines relative to A2780 cells. *p ≤ 0.05 by Student's t-test. N = 3; Mean ± SD. **C.** Survival analysis of HGSOC patients based on p53 status. The Log-rank test was used to determine statistical significance; *p* ≤ 0.05. **D**. Analysis of predicted functional activity of p53 missense mutants found in HGSOC patients. The numbers of target gene promoters not responsive to mutant p53 are shown. The percentages of total p53 mutants failing to activate the specific number of target gene promoters are shown.

The sensitive and resistant tumor models were evaluated for cytotoxic response to cis-Pt using the IC_50_ parameter to enable comparison. This evaluation indicated that A2780 cells were sensitive to cis-Pt, as indicated by a low IC_50_ value of 0.30 μM (Figure 1A). However, all four resistant cell lines gave significantly higher IC_50_ values and were, therefore, confirmed as cis-Pt resistant. The level of resistance relative to A2780 cells varied, ranging from 11-fold in HEY cells to 30-fold in OVCAR-10 cells (Figure 1B). This indicated that cis-Pt resistance was expressed in tumor models irrespective of p53 functional status by FASAY. However, it was important to examine the relevance of these models to the clinical situation. Thus, the TCGA database on ovarian serous cystadenocarcinoma (HGSOC), which is primarily treated with cis-Pt-based chemotherapy [4], was examined for patient survival. The results with a larger population cohort confirm a previous report [24] that overall survival (OS) tends toward being shorter in patients with cancers harboring wild-type p53 (median OS, 34 *vs*. 40 months; Figure 1C). Since this may be due to either dysfunctional wild-type p53 or functional mutant p53, we correlated actual p53 mutations in HGSOC cancers to functionality as mined from the p53.fr database. Our findings demonstrate that of 175 missense p53 mutants in TCGA database, only one (0.6%) was fully active against all eight p53 target gene promoters in the FASAY system, with three (1.7%) inactive against 3-4 promoters, 19 (11%) inactive against 5-6 promoters, and 152 (87%) were inactive against 7-8 promoters (Figure 1D). These results indicate that the 2780CP/Cl-16 and OVCAR-10 tumor models with non-functionality of p53 mutants are consistent with majority (98%) of p53 mutants in HGSOC.

**Figure 2:**
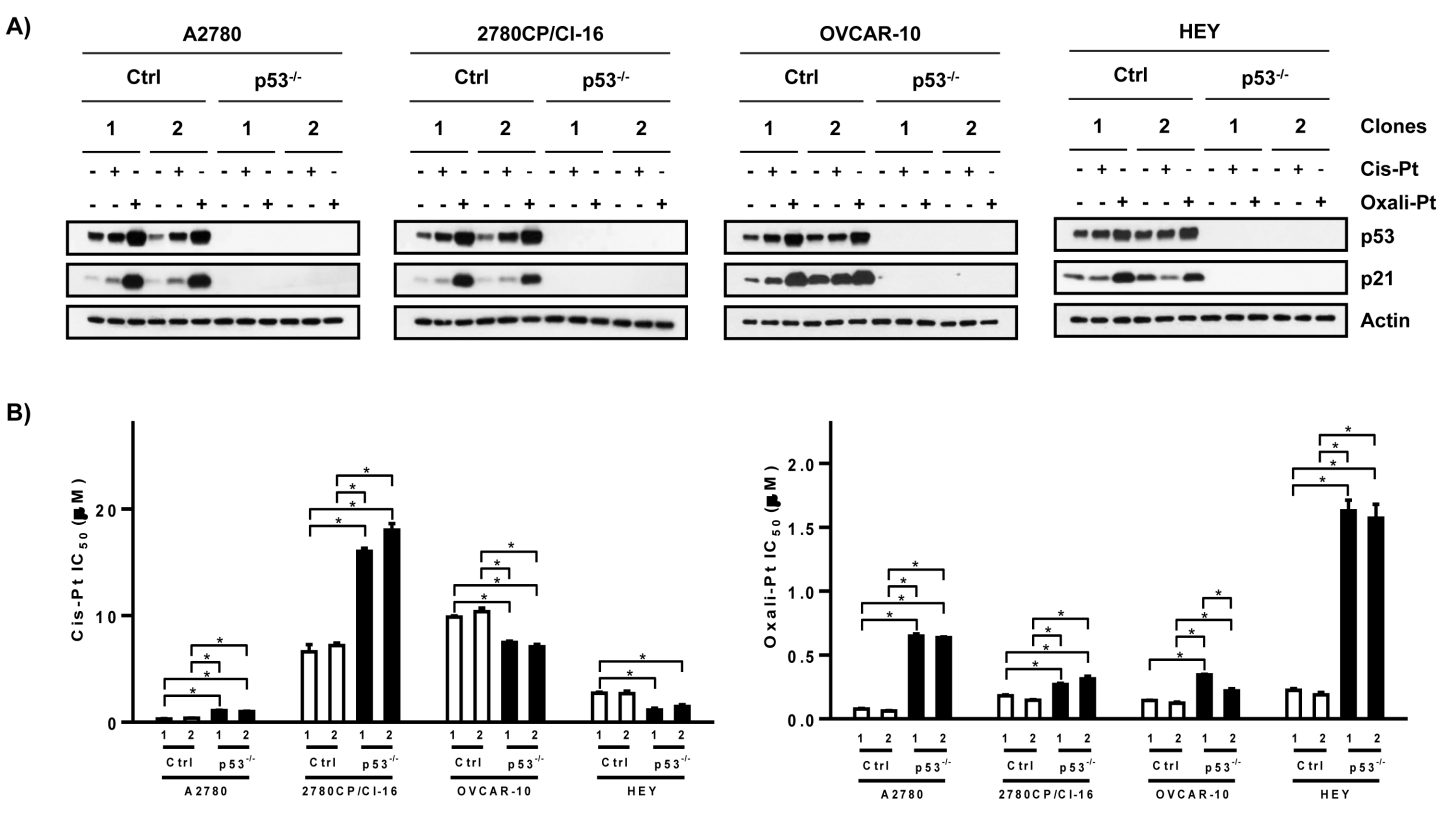
Dependence of cytotoxic platinum drug response on p53 in ovarian cancer cell lines **A.** Control (Ctrl) and p53-knockout (p53^−/−^) clones from ovarian tumor cells were exposed to cis-Pt or oxali-Pt for 24 hr and levels of p53 and p21 were examined by Western blot analysis. **B**. IC_50_ values for cis-Pt or oxali-Pt in control (Ctrl) and p53-knockout (p53^−/−^) clones was determined by the 5-day MTT assay. *p ≤ 0.05 by ANOVA. N = 3; Mean ± SD.

Interestingly, oxali-Pt was highly potent against A2780 cells (IC_50_, 0.15 μM) and against all cis-Pt-resistant cell lines, resulting in relatively low resistance factors of 1.4-3.8 (Figure 1A and 1B). Thus, oxali-Pt is able to circumvent 65-94% of resistance in cis-Pt-resistant tumor models, irrespective of p53 gene (by sequencing) or functional (by FASAY) status. To determine whether the Pt drugs were p53-dependent, clones were selected from cell lines after mock or p53 knockout and characterized. Several clones were obtained from A2780 cells, but resistant cells inexplicably yielded only few clones, with OVCA-433 cells failing to yield any clones (data not shown). For immunoblot analysis, clones were exposed to drug concentrations based on IC_50_ derived from sensitive A2780 cells; this avoided exposures of cells to supra-pharmacologic drug concentrations. Thus, A2780 clones were exposed to 1 μM cis-Pt or 0.6 μM oxali-Pt, and clones from resistant cells were exposed to 5-fold higher concentrations in order to compensate in part for lower drug uptake in resistant cells [26]. Both drugs induced p53 and p53-dependent target p21 in control A2780 clones; however, oxali-Pt expressed greater potency in inducing these proteins (Figure 2A). Interestingly, oxali-Pt induced p53 and p21 in resistant clones also, but cis-Pt had little or no effect on p53 and p21, and this is particularly evident in control HEY clones. Nevertheless, p53 dependence for drug-mediated induction of p21 can be surmised from a comparison of p21 expression between control clones and p53 knockout (p53^−/−^) clones. Significantly, it is readily evident from results with oxali-Pt that both wild-type and mutant p53 in resistant models can be consistently activated to upregulate p21.

To examine if cytotoxicity was also p53-dependent, IC_50_ was determined in p53^+/+^ (control) and p53^−/−^ clones. A 2- to 3-fold increase in IC_50_ for cis-Pt was demonstrated in A2780 and 2780CP/Cl-16 p53^−/−^ clones, but a surprisingly significant 1.4- to 2.1-fold decrease in cis-Pt IC_50_ in p53^−/−^ clones derived from OVCAR-10 and HEY cells was noted (Figure 2B). This suggests differences in cell context for the mechanism involved in dysfunctional p53 response to cis-Pt. However, in contrast, loss of p53 consistently increased IC_50_ of oxali-Pt in p53^−/−^ clones from all cell lines by 2- to 11-fold. These cytotoxic data with oxali-Pt indicating p53 dependency are in concordance with p53-dependent upregulation of p21, and strongly suggests that p53 in all cell lines is activated by this Pt analog to induce antitumor response, irrespective of p53 gene status.

### Ser20 phosphorylation of p53 enhances its transcriptional activity

**Figure 3:**
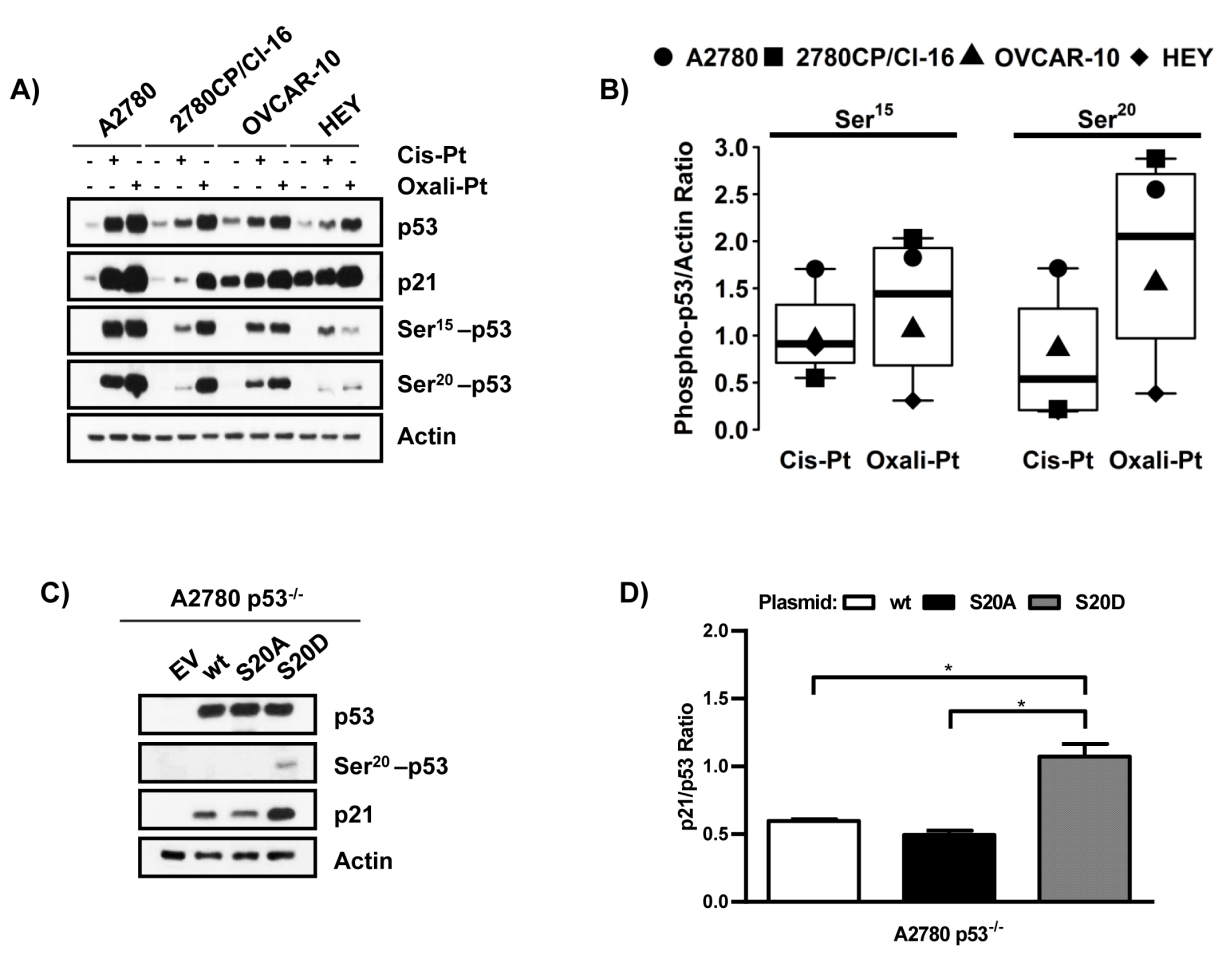
**Phosphorylation of p53 at Ser20 enhances its transcriptional activity A. Relative induction of p53, p21, p53-Ser15 or p53-Ser20 in cells exposed to 5 μM cis-Pt or oxali-Pt for 24 hr**. **B**. Quantification of p53 phosphorylation at Ser15 and Ser20 in A2780, 2780CP/Cl-16, OVCAR-10 and HEY cell lines treated with 5 μM of cis-Pt or oxali-Pt for 24 hr. **C**. Expression of p53, p53-Ser20 and p21 in A2780 p53^−/−^ cells transfected with pcDNA3 control (1 µg), wild-type p53 (0.5 µg), mutant p53-S20A (1 µg) or p53-S20D (0.5 µg) expression vectors for 48 hr. **D**. Quantification of p21 levels expressed by wild-type p53, p53-S20A or p53-S20D in A2780 p53^−/−^ cells. *Student's t-test was used to determine statistical significance at *p* ≤ 0.05. N = 3; Mean ± SD.

Given that p53 in resistant cell lines may not be activated by cis-Pt as efficiently as by oxali-Pt (Figure 2A), and that phosphorylation plays a critical role in regulating p53 function [12], the differential ability of these Pt compounds to induce p53 transcriptional activity was examined at the level of post-translational modification. Specifically, we assessed phosphorylation of p53 at Ser15 and Ser20 sites that have been reported as most critical for its anti-proliferative and pro-apoptotic functions [16, 30]. Exposure of A2780 cells to cis-Pt and oxali-Pt again demonstrated robust inductions of p53 and p21 that were associated with correspondingly robust phosphorylation of Ser15 and Ser20 (Figure 3A). In resistant cell lines, inductions of p53 and p21 by cis-Pt were again attenuated in general, whereas oxali-Pt-mediated inductions were substantially greater. This is particularly apparent when comparing results from A2780 cells and the related 2780CP/Cl-16 cells that also correlated directly with differential Ser15 or Ser20 phosphorylation by the two drugs. In OVCAR-10 and HEY cells, however, Ser15 phosphorylation by cis-Pt was similar to or greater than that by oxali-Pt, whereas Ser20 phosphorylation by oxali-Pt was consistently greater than by cis-Pt. These differences are readily apparent in Figure 3B where densitometry of phospho-bands demonstrate that the ratio of oxali-Pt:cis-Pt is ~4 for mean Ser20 and ~1.5 for mean Ser15. Moreover, induction of Ser20 phosphorylation, but not Ser15 phosphorylation, correlated directly with p53 and p21 inductions in each cell line and for each drug (Figure 3A). These results suggest that oxali-Pt efficiently induces Ser20 phosphorylation and activates p53 function in cis-Pt-resistant cells. To validate p53-Ser20 phosphorylation as enhancing its transcriptional activity, we monitored p21 levels by immunoblot in A2780 p53^−/−^ cells transfected with plasmids expressing wild-type p53, mutant p53-S20A (constitutively dephosphorylated mimic) or mutant p53-S20D (constitutively phosphorylated mimic). The results in Figure 3C and 3D do indeed demonstrate that expression of p53-S20D, which as anticipated is detectable by Ser20-p53 antibody, increases p21 to significantly greater levels (~2 fold) as compared to wild-type p53 or mutant p53-S20A. These results corroborate that Ser20 phosphorylation is an important potentiator of p53 transcriptional activity.

### Chk2 mediates cis-Pt-induced p53 transcriptional activation and cis-Pt sensitivity

**Figure 4:**
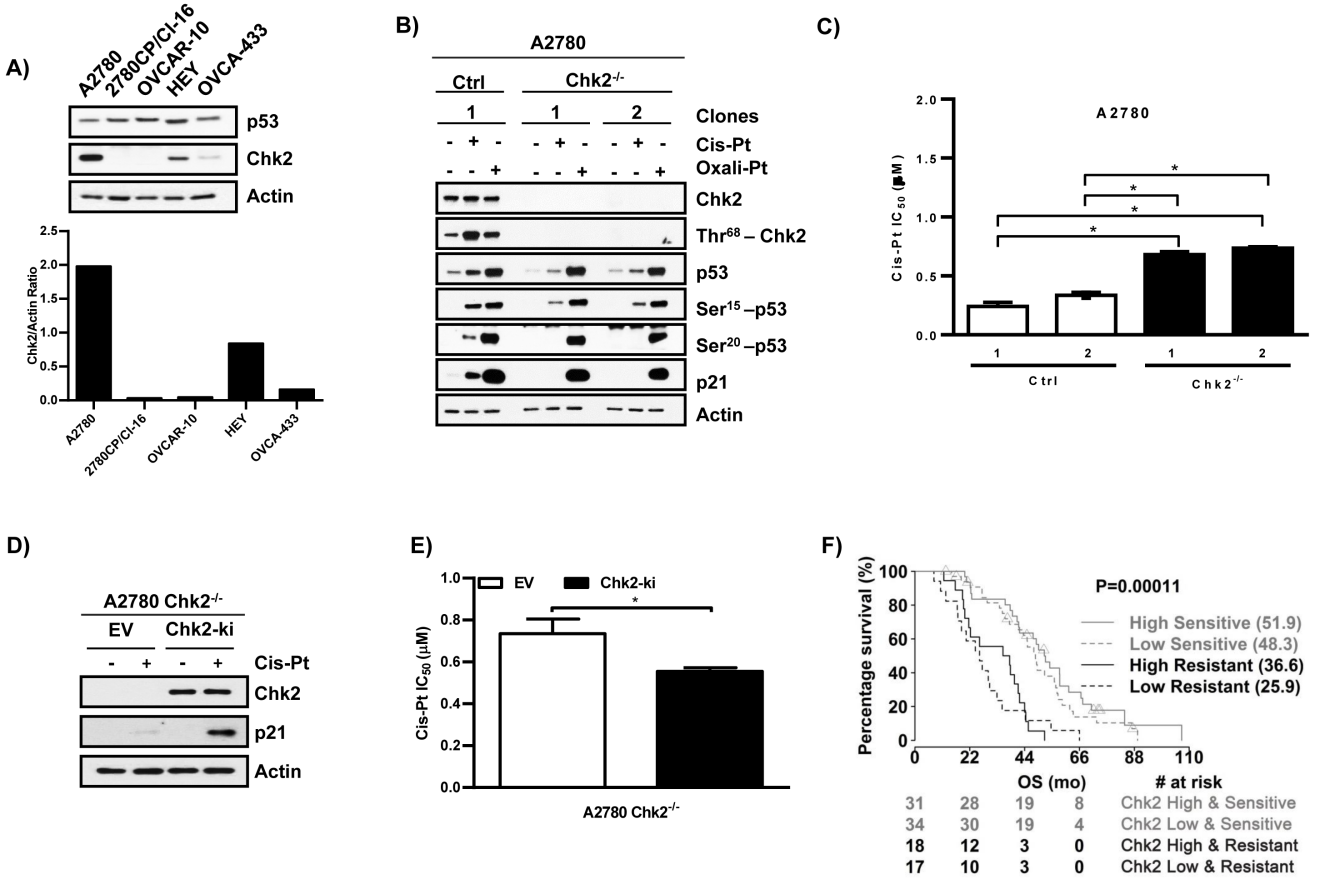
Chk2 mediates cis-Pt-induced p53 transcriptional activation and Pt sensitivity **A**. Basal levels of Chk2 protein in cell lines. **B**. Control (Ctrl) and Chk2-knockout clones from A2780 cells were exposed to cis-Pt (1 µM) or oxali-Pt (0.6 µM) for 24 hr. Proteins were examined by Western blot. **C**. Cytotoxicity of cis-Pt was assessed in control (Ctrl) and Chk2-knockout clones from A2780 cells using the 5-day MTT assay. *ANOVA test was used to determine statistical significance at *p* ≤ 0.05. N = 3; Mean ± SD. **D**. A2780-Chk2^−/−^ cells were transfected with 2 µg of pEGFP-C1 empty vector (EV) control or Chk2 (Chk2-ki) expression vectors for 48 hr. Cells were treated with cis-Pt 1µM for 24 hr and processed for Western blot analysis. **E**. Cytotoxicity of A2780 Chk2^−/−^ cells to cis-Pt following transfection with 2 µg of pEGFP-C1 control (EV) or Chk2 (Chk2-ki) expression vector for 24 hr. IC_50_ values were determined using the 3-day MTT assay protocol. *Student's t-test was used to determine statistical significance at *p* ≤ 0.05. N = 3; Mean ± SD. **F**. Overall survival of HGSOC patients based on their Chk2 expression levels (high *vs*. low) in pt sensitive or resistant cohorts. The Log-rank test was used to determine statistical significance, *p* value ≤ 0.05.

With attenuation of Ser20 phosphorylation by cis-Pt defined as a key event in cis-Pt resistance, it becomes important to understand the underlying basis for this observation. Pt-induced DNA damage triggers p53 phosphorylation *via* the activation of specific kinases [7, 31]. Studies have shown that downregulation of Chk2, a kinase reported to phosphorylate p53 at Ser20 after cis-Pt treatment, leads to resistance [32–34]. In addition, our lab has previously reported that knockdown of Chk2 in A2780 cells reduced the ability of cis-Pt to induce p53 and p21 [35]. Therefore, we evaluated basal levels of Chk2 by immunoblot across ovarian cancer cell lines. As anticipated, Chk2 was expressed in sensitive A2780 cells, but protein levels were substantially lower by 60-98% in all cis-Pt resistant cell lines (2780CP/Cl-16, OVCAR-10, HEY and OVCA-433) (Figure 4A). To validate decreased levels of Chk2 as contributing to attenuated p53-Ser20 phosphorylation and transcriptional activity, we generated A2780 Chk2-knockout (Chk2^−/−^) clones by CRISPR/Cas9. Cis-Pt or oxali-Pt treatment of A2780 control clone demonstrated that total Chk2 levels were unaffected and that induction of p53, phospho-p53 and p21 levels were consistent, and confirmed our previous report [35] that Chk2 was activated *via* Thr68 phosphorylation by cis-Pt, and to a lesser extent by oxali-Pt (Figure 4B). Chk2 knockout, on the other hand, resulted in a dramatic decrease in p53-Ser20 phosphorylation and p21 levels with cis-Pt. Remarkably, loss of Chk2 did not affect the ability of oxali-Pt to induce p53-Ser20 phosphorylation and p21 expression. Similar results were obtained by Chk2 downregulation by siRNA in A2780 cells (data not shown). To study the effect of Chk2 in cytotoxic response, the IC_50_ was determined in A2780 control and Chk2^−/−^ clones. Loss of Chk2 led to a significant increase (~2- to 3-fold) in cis-Pt IC_50_ and, therefore, cis-Pt-resistance (Figure 4C). Conversely, ectopic re-expression of Chk2 in A2780 Chk2^−/−^ clones restored cis-Pt-induced p53 transcriptional activity (Figure 4D) and cytotoxic sensitivity (IC_50_: control, 0.74 *vs*. Chk2-ki, 0.55 μM) (Figure 4E). To assess the clinical significance of Chk2 in modulating chemosensitivity, we investigated the effect of Chk2 expression in Pt therapy outcomes in HGSOC patients. For this study, we accessed the TCGA data bank, and Pt sensitive or resistant patients were clustered into two groups according to their Chk2 expression (high *vs*. low). In either group, Pt-sensitive patients with relatively high levels of Chk2 were found to have significantly greater overall survival by 4-11 months (Figure 4F). However, the difference between the drug-sensitive cohort expressing high Chk2 and drug-resistant patients expressing low Chk2 was greater (26 months). Taken together, these results indicate that Chk2 is essential for cis-Pt-induced p53-Ser20 phosphorylation and p53 transcriptional activity, and is an important modulator of cis-Pt chemosensitivity.

### Oxali-Pt induces p53-Ser20 *via* MEK1/2

**Figure 5:**
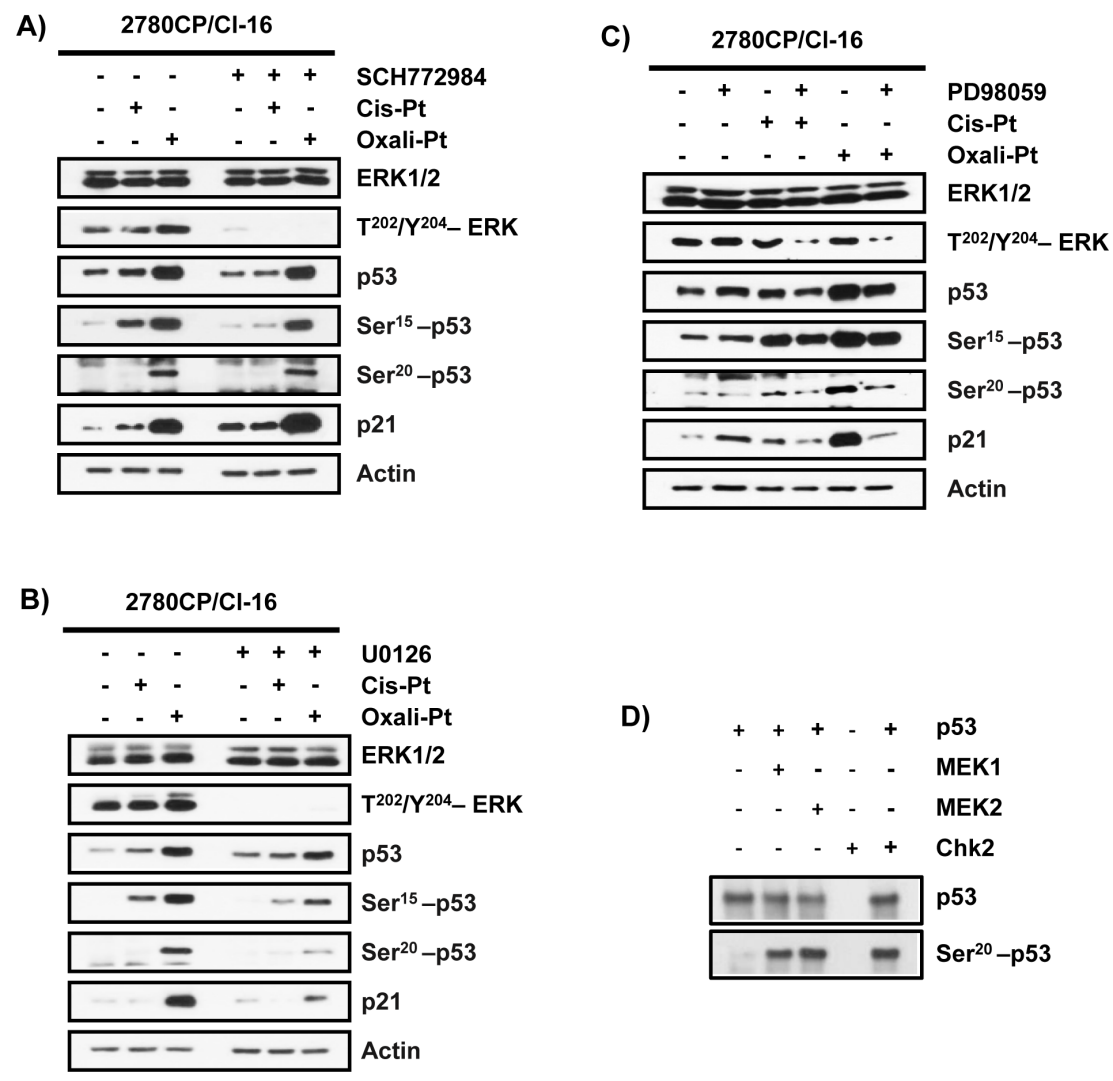
Modulation of phosphorylation of p53 by ERK and MEK inhibitors The effect of **A**. SCH772984 (1.5 µM; 1 hr), **B**. U0126 (10 µM; 1 hr), and **C**. PD98059 (100 µM; 1 hr) in 2780CP/Cl-16 cells on levels of indicated proteins induced by cis-Pt (5 µM) and/or oxali-Pt (3 µM) at 24 hr. **D**. Western blot analysis of phosphorylation of recombinant p53 at Ser20 by active recombinant MEK1 and MEK2 and immunoprecipitated Chk2 (positive control) from cis-Pt-treated A2780 cells.

In this study, oxali-Pt has demonstrated the capacity to restore p53-Ser20 phosphorylation and p53 transcriptional activity independently of Chk2, but the kinase involved in this Ser20 modification is not known and requires attention. We have previously demonstrated that p53-dependent induction of p21 by an oxali-Pt-like analog DACH-diacetato-dichloro-Pt(IV) was inhibited by the PI3-K inhibitor wortmannin [14]. However, PI3-K is not known to phosphorylate p53, but wortmannin can also inhibit MAPKs [36], which may then impact p53 function. Specifically, MAPK members ERK1/2 and MEK1/2 can transcriptionally activate p53 [37, 38] and MEK inhibitor U0126 is reported to induce cis-Pt resistance [39]. Based on this collective evidence, phosphorylation of Ser20 by MAPK can be rationalized. For this reason, we explored ERK1/2 and MEK1/2 inhibitors to provisionally examine their potential to phosphorylate p53 at Ser20. However, treatment of 2780CP/Cl-16 cells with the ERK1/2-selective ATP-competitive inhibitor (ERKi) SCH772984 did not impact the ability of oxali-Pt to induce p53-Ser20 phosphorylation or p21 expression, even though it inhibited phospho-ERK and Ser15 phosphorylation by cis-Pt (Figure 5A). On the other hand, oxali-Pt-induced p53-Ser20 phosphorylation and p21 transactivation in 2780CP/Cl-16 cells were inhibited by the selective MEK1/2 inhibitors (MEKi) U0126 and PD98059 (Figures 5B and 5C). Since MEK1/2-mediated p53-Ser20 phosphorylation is not readily evident from the literature, we investigated the potential of this kinase to phosphorylate p53-Ser20 directly. We included Chk2 immunoprecipitate isolated from A2780 cells treated with cis-Pt as a positive control. Indeed, active recombinant MEK1, MEK2 and Chk2 were able to phosphorylate p53 at the Ser20 site (Figure 5D). This demonstrates for the first time, to our knowledge, that MEK1/2 has the potential to directly phosphorylate p53 at the Ser20 site.

## DISCUSSION

The p53 pathway when functionally activated through post-translational modifications plays a major role in mediating Pt chemotherapy response. Therefore, it is not surprising that loss of p53 function through mutation has been identified as an important mechanism leading to cis-Pt resistance [20, 31]. In HGSOC, the high p53 mutation rate of 86%, based on TCGA data shown in Figure 1C, presents a major therapeutic barrier. In this study, however, we have demonstrated that the four ovarian cancer cell lines resistant to cis-Pt, irrespective of p53 genotype, were responsive to oxali-Pt and demonstrated low cross-resistance to this Pt analog. This cytotoxic difference was due to the relative abilities of the two drugs to functionally activate p53, with cis-Pt having an attenuated or negligible effect and oxali-Pt demonstrating a robust effect. The poor cis-Pt-dependent cytotoxicity and lack of p53 activation was due to downregulation of Chk2 and resultant reduction in Ser20 phosphorylation, a key event normally required to stabilize and activate p53. The significance of Chk2 downregulation in cis-Pt resistance was confirmed in HGSOC by reduced overall survival in patients. This is consistent with a recent report that Chk2 is a good biomarker for Pt chemotherapy in advanced stage HGSOC patients [34]. Oxali-Pt, on the other hand, was not dependent on Chk2 in our study and activated p53 *via* an independent kinase, which appears to be MEK1/2. Although Chk2 activated by cis-Pt has been reported to phosphorylate Ser20 [33] and its knock-down in A2780 cells reduces the ability of cis-Pt to induce p21 in a p53-dependent manner [35], MEK1/2 has not been associated previously with phosphorylation at Ser20. Therefore, this regulation of p53 by MEK1/2 is a novel observation.

**Figure 6:**
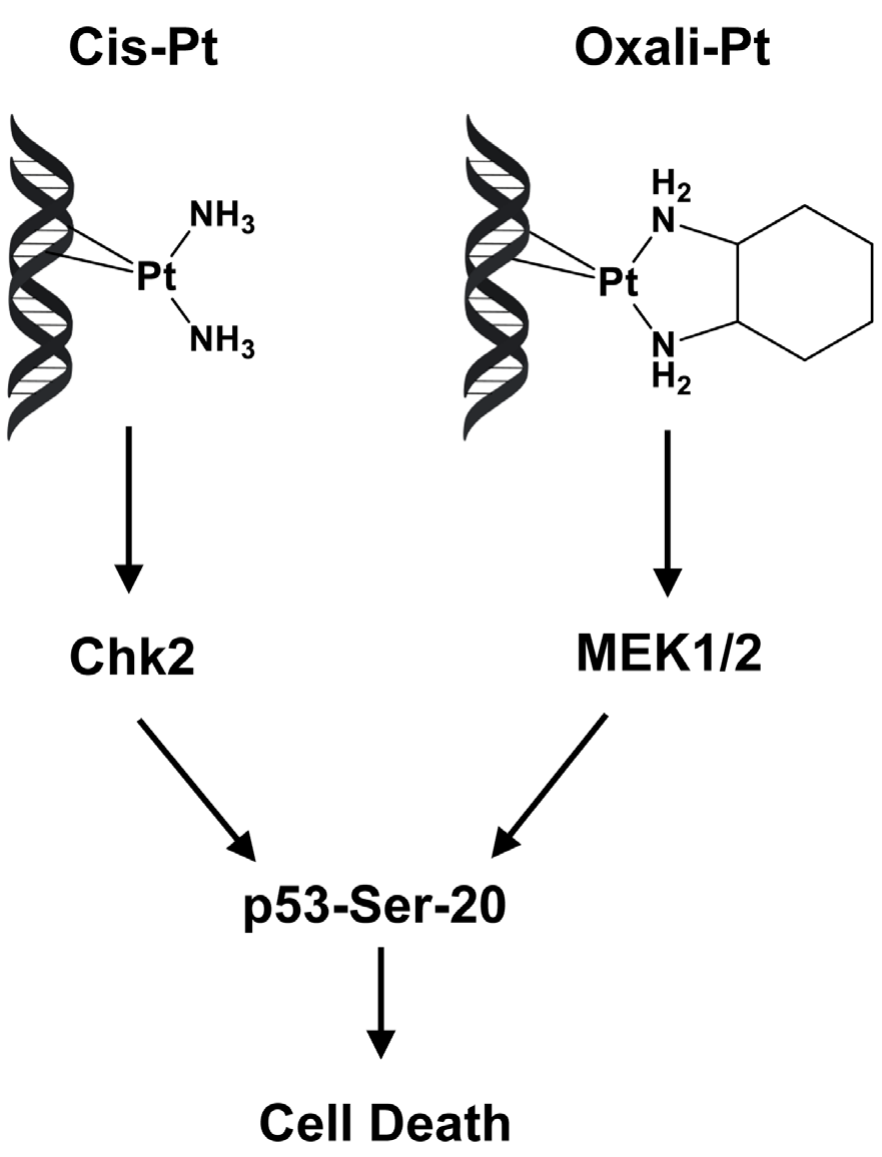
Model for independent pathways involved in phosphorylation of p53 at Ser20 by cis-Pt and oxali-Pt By virtue of structural differences, cis-Pt and oxali-Pt intrastrand adducts transduce DNA damage signal along distinct pathways that converge to phosphorylate p53 at Ser20, activate p53 and induce cell death. When Chk2 is downregulated, p53 activation by cis-Pt is inhibited and, therefore, resistance to this drug is affected. However, oxali-Pt can restore p53 function and circumvent cis-Pt resistance by activating the MEK1/2 kinase.

The differential effect of cis-Pt and oxali-Pt in functionally activating p53 in resistant cells is consistent with the two Pt-based drugs having independent mechanisms of action. Although both drugs target DNA and form identical intrastrand crosslinks, primarily G-G adducts, the effects of these adducts are distinct due to structural differences, as shown in Figure 6. Biochemical studies have demonstrated that the structural variation between cis-Pt and oxali-Pt adducts is sufficient to induce significant differences in the degree of DNA bending and unwinding [40]. These drug-dependent local distortions in damaged DNA are, therefore, recognized independently by specialized proteins, such as specific members of the high mobility group box (HMGB) and mismatch repair (MMR) families that bind only cis-Pt-DNA adducts, but not oxali-Pt adducts [41]. The specificity of recognition proteins likely dictates upregulation of the individual kinase involved in activation of p53 function (Figure 6). This understanding is consistent with the multiplicity of kinases involved in phosphorylation of individual amino acid residue, including Ser20, for p53 regulation [7], and has also been demonstrated in Ataxia Telangiectasia (AT) cells, which have defective ATM kinase. Thus, p53 in AT cells is not responsive to DNA strand breaks by ionizing radiation due to loss of ATM kinase, but its regulation is restored by DNA adducts induced by ultraviolet radiation or cis-Pt that instead activate the intact, closely-related ATR kinase [42, 43].

Transcriptional activity of p53 is inhibited by its binding to Mdm2 and Mdm4, but is essential for its function, which is affected *via* post-translational phosphorylations that disrupt the Mdm2-Mdm4-p53 complex and release p53 [7]. Although several sites in p53 are amenable to phosphorylation following DNA damage, modifications at Ser15 and Ser20 are reported as most critical for its anti-proliferative and apoptotic functions [15–17]. However, in our present study, p53-Ser20 phosphorylation appeared to correlate consistently with p21 transactivation and drug cytotoxicity in all cell lines. Since Mdm2 has E3 ligase activity and Mdm4 is reported to inhibit p53 transactivation [44–46], it is possible that Ser15 phosphorylation is significant for disrupting Mdm2 binding and/or E3 ligase activity to promote p53 stabilization, with Ser20 phosphorylation becoming important for disrupting Mdm4 binding that then enables p53 to interact with p300 coactivator, which is required for p53 transactivation function [17, 45, 47]. This is in agreement with the report that Ser15 phosphorylation is more effective in stabilizing p53 [48].

An important discovery in our study was the substantial cis-Pt resistance in the four cell lines whether they harbored wild-type (polymorphic) or mutant p53. The fact that both wild-type or mutant p53 in these resistant cells was functionally activated by oxali-Pt indicates that V172F and G266R mutations found in 2780CP/Cl-16 and/or OVCAR-10 cells were not inactivating, although the p53 functional FASAY database predicted these p53 mutants as inactive. The reason for this discrepancy is not known, but it is possible that Ser20 phosphorylation by oxali-Pt may alter the p53 configuration in a manner that restores its transactivation function in human tumor cells. However, this may not entirely be the case since our recent report has demonstrated that the V172F-p53 mutant can upregulate p21 in absence of Ser20 phosphorylation when 2780CP/Cl-16 cells are challenged with the MDM2 inhibitor nutlin-3 [49]. Our data nonetheless indicate that the specific mutations *per se* were not the inactivating event, but rather the loss of Ser20 phosphorylation due to downregulation of Chk2 in the four cell lines was the primary driver of resistance of the cell lines to cis-Pt. This is consistent with reduced response rate [34] or reduced overall survival (this study) in HGSOC patients as a result of low Chk2 expression. The functional competency of the specific p53 mutants in our study is also consistent with a reported detailed analysis demonstrating that of 2,314 distinct missense p53 mutants, almost 64% retained p53 activity comparable to that of wild-type p53 [25]. This raises the intriguing possibility that some of the p53 mutants in HGSOC that were identified as functionally inactive from our FASAY databank mining may in fact be functional. In a similar manner, the data with HEY and OVCA-433 suggests that cis-Pt resistance in clinical tumors harboring polymorphic/wild-type p53 may also be at the level of a defect in post-translational phosphorylation at Ser20.

In summary, our present study demonstrates that Ser20 phosphorylation of p53 dictates cytotoxic response to cis-Pt and oxali-Pt, and loss of this post-translational phosphorylation in absence of Chk2 prevents p53 activation by cis-Pt and this leads to drug resistance. However, cis-Pt resistance can be circumvented by oxali-Pt *via* activation of MEK1/2, which restores Ser20 phosphorylation of p53. More significantly, Ser20 phosphorylation of specific p53 mutants with oxali-Pt also activates p53 function, and this raises the likelihood that other p53 mutants in refractory human ovarian cancers could also be functionally activated with distinct drugs, such as oxali-Pt, to restore therapeutic sensitivity.

## MATERIALS AND METHODS

### Cell culture

The A2780 cell line was derived from a drug-naïve patient and is considered to be sensitive to cis-Pt [50]. The 2780CP/Cl-16 cell line was derived as a clone from A2780/C30 cells, which were made cis-Pt-resistant by intermittent exposure to cis-Pt [26]. A2780 and A2780/C30 cell lines were kindly provided by Dr. Thomas Hamilton (Fox Chase Cancer Center, Philadelphia, PA, USA). The source of OVCAR-10, HEY and OVCA-433 cell lines and their maintenance in culture have been described previously [51]. All cell lines were negative for mycoplasma and authenticated by short tandem Q2 repeat DNA fingerprinting in MD Anderson Core Facilities. All cell lines were grown in an atmosphere of 37°C and 5% CO_2_.

### Antibodies and reagents

Antibodies specific to p53 (1:2000 dilution) (sc-126), p21 (1:500) (sc-6246), β-actin (1:4000) (sc-47778), Ser20-p53 (1:500) (sc-18079-R) and Chk2 (1:500) (sc-9064), were purchased from Santa Cruz Biotechnology. Antibodies against Ser15-p53 (1:1000) (9284), Thr68-Chk2 (1:1000) (2197), ERK1/2 (1:1000) (4695) and T202/Y204-ERK (1:1000) (4376) were purchased from Cell Signaling. Secondary ECL Anti-Mouse IgG, Horseradish (1:4000) (from sheep) (NA931) and ECL Anti-Rabbit IgG, Horseradish (1:4000) (from sheep) (NA934) were purchased from GE Healthcare. The ERK- and MEK-specific inhibitors, SCH772984 (S7101) and U0126 (S1102), respectively, were purchased from Selleckchem Chemicals. The specific MEK inhibitor PD98059 (9900L) was purchased from Cell Signaling. All inhibitors were diluted in DMSO and stored in aliquots at −20°C. MTT (298-93-1) was purchased from Fisher Scientific. Clarity™ Western ECL Substrate (1705061) and 4-15% Mini-PROTEAN^®^ TGX™ Precast Protein Gels (4561086) were obtained from Bio-Rad.

### p53 sequencing

A pellet corresponding to 1 × 10^6^ cells was washed with ice cold PBS, and DNA extracted using the QIAamp^®^ DNA Mini Kit (QIAGEN) following the manufacturer's protocol. Extracted DNA was quantified using the NanoDrop (Thermo Fisher Scientific). DNA samples were submitted for *TP53* sequencing to the Sequencing and Microarray Core Facility at The University of Texas MD Anderson Cancer Center, Houston, TX.

### Cytotoxic evaluations

The procedure employed for cytotoxic evaluations was adapted from our previous report [51]. Ovarian cancer cells growing in tissue culture dishes were trypsinized, diluted to appropriate concentrations, and 100 μL/well aliquots plated in 96-well plates to achieve the following densities: A2780, 200 cells/well; 2780CP/Cl-16, 500 cells/well; OVCAR-10, 500 cells/well; HEY, 150 cells/well; and OVCA-433, 300 cells/well. Plates were incubated overnight at 37°C. Aliquots of 100 μL serially-diluted solutions of Pt drugs in medium were then added to ach well. Plates were further incubated at 37°C for 5 days. After this time, 50 μL of an MTT solution (3 mg/mL) was added to each well and plates were incubated for 4 hr. The medium was removed and purple MTT formazan crystals were dissolved in 100 μL 100% DMSO. Plates were shaken for 5-10 min and absorbance measured at 570 nm with a multiwell scanning spectrophotometer (Molecular Devices, Sunnyvale, CA, USA). IC_50_ values were determined from the sigmoidal plot of % cell survival *vs*. drug concentration using commercial software (GraphPad Prism v.6; La Jolla, CA, USA).

### Transfections

The p53 plasmids were kindly provided by Dr. Mickey C.-T. Hu (Stanford University School of Medicine, Stanford, CA, USA). A 0.5 µg of p53, 1 µg of S20A or 0.5 µg of S20D plasmid aliquot was transfected into A2780-p53^−/−^ cells in 6-well plates using Lipofectamine 2000 (Life Technologies) for 48 hr, following the manufacturer's recommended protocol. The Chk2 plasmid (2 µg), a gift from Dr. Junjie Chen (The University of Texas MD Anderson Cancer Center, Houston, TX, USA), was similarly transfected into A2780-Chk2^−/−^ cells. After 48 hr transfection, cells were treated with Pt drugs and collected 24 hr later for immunoblot analysis. For IC_50_ analysis, cells were trypsinized after 24 hr of transfection and were subjected to the above described cytotoxic MTT evaluation procedure using a 3-day drug exposure protocol.

### Generation of stable gene-knockout clones by CRISPR

CRISPR plasmids *TP53* (HS0000019748, NM_001126117), *CHEK2* (HS0000041294, NM_001005735) and universal negative control (CRISPR08) were purchased from Sigma-Aldrich. Cells at 70% confluence in T75 flask were transfected with 25 μg of CRISPR/Cas-GFP plasmid using Lipofectamine 2000 (Life Technologies) following the manufacturer's recommended protocol. After 48 hr transfection, GFP positive cells were sorted using the BD FACSAria™ cell sorter (BD Biosciences), collected and grown in T75 flasks. At 80% confluence, cells were trypsinized and diluted aliquots of 100, 200, 300, 400 and 500 cells were grown in 10-cm dishes. Single clones from the 10-cm dishes were selected, grown in 12-well plates and characterized for knockout through Western blot analysis.

### Western blot analysis

Cells were scraped and centrifuged at 3,000 rpm and 4°C for 1 min. Cell pellets were resuspended and sonicated in 50-100 µL ice-cold extraction buffer (50 mM Tris HCl; pH 7.4), 10 mM NaF, 2 mM EDTA, 150 mM NaCl) with 0.5% NP-40 and 2 mM phosphatase inhibitors. Cell lysates were centrifuged at 15,000 rpm at 4°C for 10 min. Supernatants were isolated, quantified for protein concentration by BCA assay and processed for immunoblotting. A 50 μg aliquot of protein extracts were run on a 4-15% gradient SDS-PAGE ready gel, electrophoretically transferred for 1 hr at 300 mA to a nitrocellulose membrane, blocked with 5% milk for 1 hr, and probed with primary antibody overnight and secondary antibody for 1 hr. Finally, blots were developed by chemiluminescence. For densitometric analysis of the band, the X-ray films were scanned and the signals were analyzed using the Image J software.

### Phosphorylation at Ser20-p53 by MAPK

2780CP/Cl-16 cells were seeded into 6-well plates and incubated overnight at 37°C. Cells were then treated for 1 hr with DMSO, 1.5 µM of ERK1/2 inhibitor SCH772984, 100 µM of MEK1/2 inhibitor PD98059 or 10 µM of the MEK1/2 inhibitor U0126. After 1 hr, cells were treated with 5 μM cis-Pt or 3 μM oxali-Pt and collected 24 hr later for immunoblot analysis.

For MEK-mediated phosphorylation, 25 μM of ATP (Sigma-Aldrich), 12.5 ng/μL of recombinant p53 (Santa Cruz Biotechnology) and 20 ng/μL of active recombinant MEK-1 or MEK-2 (SignalChem) were added in a 1.5 mL microfuge tube. Immunoprecipitated Chk2 from A2780 cells treated with cis-Pt 1 μM was used as a positive control. Chk2 immunoprecipitate was mixed with 25 μM of ATP and 12.5 ng/μL of recombinant p53. Reaction Buffer (150 mM NaCl, 4 mM MnCl_2_, 6 mM MgCl_2_, 10% (v/v) glycerol, 1 mM dithiothreitol, 100 μM Na_3_VO_4_, 50 mM HEPES (pH 7.5)) was then added for a final volume reaction of 25 μL. The reaction was incubated at 30°C for 20 min. After this time, the mixture was examined by immunoblot analysis.

### Analysis of patient survival

The reverse phase protein array (RPPA) Level 3 data for Chk2 in patients with ovarian serous cystadenocarcinoma (HGSOC) was accessed from the TCGA (http://tcga-data.nci.nih.gov/). Overall survival information, response to Pt and TP53 mutation status were retrieved from cBioPortal (http://www.cbioportal.org/). The analyses were carried out in R (version 3.2.2). All tests were two-sided and considered statistically significant at the 0.05 level. The Log-rank test assessed association between TP53 mutation status (wild-type *vs*. mutant) and overall survival, as depicted in Kaplan-Meyer curves. Patients were grouped as platinum resistant and platinum sensitive cohorts, taking into account the classification-system based on the platinum free interval. The cohorts were further stratified into high and low Chk2 RPPA level groups at percentile cutoffs between 0.25 and 0.75 with a step of 0.01. The optimal cutoff percentile (as determined by differences in median survival time between different groups) was identified for ChK2 and Kaplan-Meier plots generated.

### Predicted functionality of mutant p53

Mutations of p53 in tumors of HGSOC patients from the TCGA database and in ovarian cancer cell lines were evaluated for their effect on p53 function using the yeast FASAY data stored in a p53 database (http://p53.fr). The mutation was assessed against eight gene promoters targeted by wild-type, and the number of promoters activated by each mutant p53 was quantified.

### Statistical analysis of experimental data

The data are expressed as mean ± standard deviation of at least three independent determinations. The statistical significance between groups was analyzed by a two-tailed unpaired Student's t test or by ANOVA. *P*-values < 0.05 were considered statistically significant.
